# Complete Response to Immunotherapy in Patients With Hepatocellular Carcinoma

**DOI:** 10.1001/jamanetworkopen.2024.61735

**Published:** 2025-02-25

**Authors:** Mir Lim, Magdalena Espinoza, Yi-Hsiang Huang, Joseph Franses, Hao Zhu, David Hsiehchen

**Affiliations:** 1 Division of Hematology and Oncology, Department of Internal Medicine, University of Texas Southwestern Medical Center, Dallas; 2 Division of Digestive and Liver Diseases, Department of Internal Medicine, University of Texas Southwestern Medical Center, Dallas; 3 Division of Gastroenterology and Hepatology, Taipei Veterans General Hospital, Institute of Clinical Medicine, Faculty of Medicine, National Yang Ming Chiao Tung University, Taipei, Taiwan; 4 Section of Hematology and Oncology, Department of Medicine, University of Chicago, Chicago, Illinois; 5 Harold C. Simmons Comprehensive Cancer Center, University of Texas Southwestern Medical Center, Dallas

## Abstract

**Importance:**

Immunotherapies are the preferred frontline treatment for most hepatocellular carcinomas (HCCs), but only a small subset of patients attain complete responses to immunotherapies, resulting in an absence of radiographic apparent disease. The clinical importance of these complete responses is unclear, and whether clinical or molecular features may define HCCs that are exquisitely sensitive to immunotherapies is ambiguous.

**Objective:**

To describe the long-term survival outcomes of complete responders to immunotherapies, and to examine whether clinical and genomic characteristics are associated with complete response.

**Design, Setting, and Participants:**

This cohort study includes a post hoc analysis of the IMbrave150 trial (which enrolled patients across 17 countries in North America, Europe, Asia, and Australia) and a multicenter cohort analysis of patients with advanced HCC treated with frontline immunotherapies across 3 institutions (2 in the US and 1 in Asia). Complete responders were defined as patients with a complete response according to Response Evaluation Criteria in Solid Tumors (RECIST) 1.1 or modified RECIST. Statistical analyses were conducted from January to May 2024.

**Main Outcomes and Measures:**

The main outcome was overall survival measured as the time from start of immunotherapy to the date of death. Kaplan-Meier curves were used to estimate progression-free and overall survival, and differences in survival outcomes were assessed using the log-rank test.

**Results:**

The analytical sample included 279 patients (mean [SD] age, 64.7 [11.1] years; 228 male patients [81.7%]) treated with atezolizumab and bevacizumab in the IMbrave150 trial and 194 patients (mean [SD] age, 64.3 [11.4] years; 155 male patients [78.7%]) with frontline anti–programmed cell death 1 ligand 1 (PD-1/L1) therapies in a multicenter patient cohort. Complete responders had high disease-free survival rates at 2 years in the IMbrave150 (58.0%; 95% CI, 36.2%-74.7%) and multicenter (87.4%; 95% CI, 58.2%-96.7%) cohorts. Overall survival rates at 2 years for complete responders were 81.1% (95% CI, 64.4%-90.5%) in the IMbrave150 cohort and 93.3% (95% CI, 61.2%-99.0%) in the multicenter cohort. Among complete responders who discontinued treatment for reasons other than disease progression after a median duration of treatment of 24 months, disease recurrence was rare. Molecular profiling of HCC among complete responders did not reveal unique genetic alterations in these patients. However, cancers with complete responses had higher PD-L1 protein expression in the immune cell compartment and lower circulating tumor DNA levels.

**Conclusions and Relevance:**

In this post hoc trial and multicenter cohort analysis of patients with HCC treated with immunotherapy, complete responders demonstrated prolonged survival and durable disease control even after discontinuation of therapy. Biological features of complete responders were also distinct, and further evaluation of immune cell PD-L1 protein expression and circulating tumor DNA as potential biomarkers is warranted.

## Key Points

**Question:**

What are the long-term survival outcomes and characteristics of patients with advanced hepatocellular carcinoma who attain a complete response while receiving immunotherapy?

**Findings:**

In this cohort study, a post hoc analysis of the IMbrave150 trial (279 patients) and a multicenter cohort analysis (194 patients) found that, in a subset of patients, complete responders to immunotherapy had prolonged overall survival and durable response while not receiving therapy. Complete responders also had greater immune cell programmed cell death 1 ligand 1 expression and lower circulating tumor DNA levels.

**Meaning:**

These findings suggest that immunotherapy complete responders are a distinct patient population with prolonged survival and specific biomarkers, and further evaluation of these biomarkers is warranted.

## Teaser

This cohort study examines the long-term survival outcomes of patients with hepatocellular carcinoma who had a complete response to immunotherapies and evaluates whether clinical and genomic characteristics are associated with complete response.

## Introduction

Hepatocellular carcinoma (HCC) is frequently a lethal cancer and remains a primary cause of cancer deaths worldwide.^1,2^ Immune checkpoint inhibitors have emerged as effective systemic treatments for HCC, and multiple immunotherapy combination regimens are approved in the first-line setting for advanced stage disease.^3,4,5^ Nonetheless, responses to immunotherapy in HCC are heterogeneous, with a minority of patients attaining some degree of tumor shrinkage and a majority of patients experiencing disease stability or progression.^4,5^ This variability in response is likely associated with several factors, including tumor genetics, the immune microenvironment, and host characteristics, although definitive biomarkers of immunotherapy sensitivity or resistance in HCC remain under investigation.^6,7^

Results from pivotal trials^4,5^ testing immunotherapies in HCC indicate that a rare population of patients can attain deep responses with no radiographic or clinical evidence of disease after treatment. The long-term outcomes of these patients have not been well-examined to date, and it is unclear whether such patients represent a clinically distinct population. In addition, investigating common characteristics of these patients may reveal novel insights into the clinical factors and biological mechanisms underlying these complete responses, but this subject remains poorly studied to date. Here, we characterize long-term survival outcomes and clinical-genomic features of complete responders to immunotherapy (defined as a complete response according to Response Evaluation Criteria in Solid Tumors [RECIST] or modified RECIST) among patients with HCC from the IMbrave150 trial and a large multicenter cohort of patients treated with anti–programmed cell death 1 ligand 1 (PD-1/L1) therapies in the first-line setting.^5^

## Methods

### Patient Cohorts

This cohort study examined patients with advanced stage HCC treated with first-line immunotherapy in the IMbrave150 trial^5^ and in an international multicenter population (not previously published). The IMbrave150 trial was a randomized phase 3 trial enrolling patients across 17 countries in North America, Europe, Asia, and Australia that demonstrated the superiority of atezolizumab plus bevacizumab vs sorafenib in patients with Child-Pugh class A disease and advanced HCC who had not been previously treated with systemic therapies. The IMbrave150 cohort in this analysis included patients treated with atezolizumab and bevacizumab in the intent-to-treat population because of legal, regulatory, or contractual constraints in data availability (eFigure in Supplement 1). Tumors were assessed in the IMbrave150 trial by computed tomography or magnetic resonance imaging at baseline and every 6 weeks until week 54 and then every 9 weeks thereafter. Data from IMbrave150 were accessed through Vivli, a public data-sharing platform. The multicenter cohort included patients with Child-Pugh class A or B disease treated in the first-line setting with anti-PD-1/L1 therapies (atezolizumab plus bevacizumab, durvalumab, durvalumab plus tremelimumab, pembrolizumab, or nivolumab) across 3 centers (2 in the US and 1 in Asia) that were not enrolling sites for the IMbrave150 trial. These patients were not allowed to have received prior anti-PD-1/L1 therapies in any setting, including neoadjuvant or adjuvant treatment. Patients were retrospectively identified after investigators received institutional review board approval at their respective institutions. Patient characteristics and clinical data were abstracted from electronic medical records. All patients in the multicenter cohort were required to have a baseline scan taken within 5 weeks of immunotherapy initiation treatment, and must have had at least 1 subsequent radiographic tumor assessment after starting immunotherapy. Tumors were assessed in the multicenter cohort by computed tomography or magnetic resonance imaging every 2 to 3 cycles of treatment (approximating 6-9 weeks). No other exclusion criterion was used to select patients for the IMbrave150 or multicenter cohorts. Complete responses were defined by RECIST as the disappearance of all lesions and by modified RECIST as the disappearance of all enhancing lesions. An institutional-determined waiver was obtained for the analysis of the IMbrave150 cohort because the dataset is deidentified and publicly available. The multicenter cohort study was approved by the institutional review board at each of the respective sites, which waived the need for informed consent because the study was retrospective and posed minimal patient risk, in accordance with 45 CFR §46. This study follows the Strengthening the Reporting of Observational Studies in Epidemiology (STROBE) reporting guidelines for cohort studies.

### Molecular Profiling

A subset of patients in the IMbrave150 cohort had DNA isolated from formalin-fixed and paraffin-embedded tumor tissue specimens that were analyzed using an assay (F1CDx; Foundation One) that uses targeted, high-throughput, hybridization-based capture technology for the detection of alterations and copy number alterations in 324 genes, and tumor mutation burden using DNA isolated from formalin-fixed and paraffin-embedded tumor tissue specimens. Tissue specimens from patients in the IMbrave150 trial were also analyzed for PD-L1 protein level by immunohistochemistry performed at a central laboratory with a PD-L1 immunohistochemistry assay (SP263; Ventana Medical Systems), and was characterized according to the combined scoring of PD-L1 protein expression on tumor cells and tumor-infiltrating immune cells. Additional details regarding the methods used to analyze the IMbrave150 cohort can be found in the primary article.^8^ Primary data from the IMbrave150 cohort are deposited in the European Genome-Phenome Archive (accession No. EGAS00001005503). A subset of patients in the multicenter cohort had peripheral blood collected before starting immunotherapy as a component of routine clinical care that was analyzed for circulating tumor DNA (ctDNA) using a US Food and Drug Administration–approved assay in a Clinical Laboratory Improvement Amendments–certified, College of American Pathologists–accredited laboratory (Guardant). The Guardant360 assay is a targeted high throughput hybridization-based capture technology for the detection of single-nucleotide variants, insertions, and deletions in 84 genes by paired-end synthesis-sequencing using the HiSeq 2500 platform (Illumina, Inc). For the pooled analysis of complete responders, only variants covered by both the Foundation One and Guardant assays were analyzed.

### Statistical Analysis

Statistical analyses were conducted from January to May 2024 using SPSS statistical software version 24 (IBM). Kaplan-Meier curves were used to estimate progression-free and overall survival, and differences in survival outcomes were assessed using the log-rank test. Associations between complete responders and other patients with clinical characteristics and molecular alterations were assessed using the χ^2^ test. Statistical significance was set at 2-sided *P* < .05. Differences in ctDNA variant allele fractions, tumor mutation burden, and PD-L1 protein expression between complete responders and other patients were assessed using the Mann-Whitney *U* test given the nonnormal distribution of data values.

## Results

**Figure 1.**
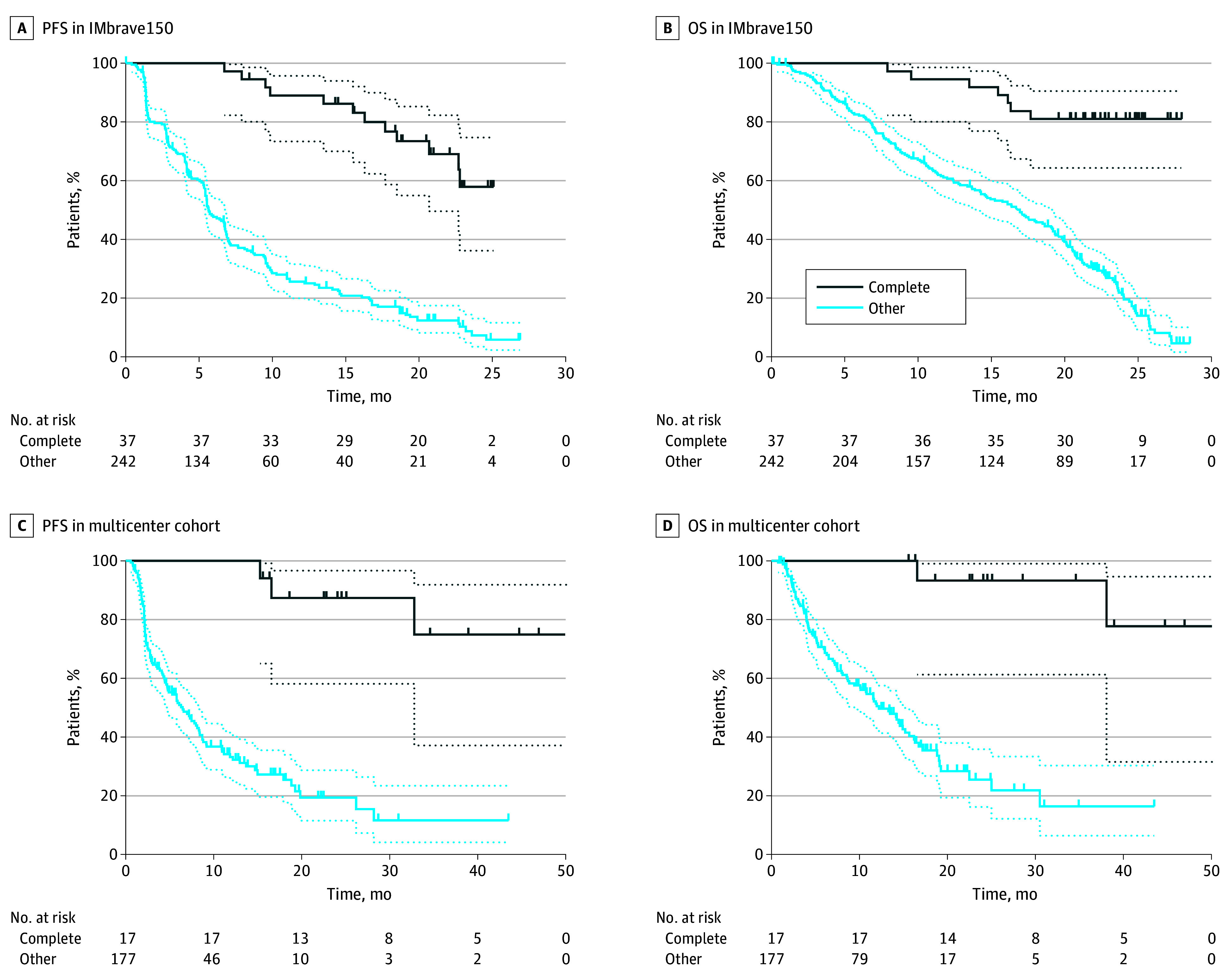
Survival Outcomes of Immunotherapy Complete Responders vs Other Patients A and B, Graphs show Kaplan-Meier analyses of progression-free survival (PFS) (A) and overall survival (OS) (B) in the IMbrave150 cohort. C and D, Graphs show Kaplan-Meier analyses of PFS (C) and OS (D) in the multicenter cohort. Dotted lines indicate 95% CIs.

Among 279 of 336 patients treated with immunotherapy in the IMbrave150 trial with available data (mean [SD] age, 64.7 [11.1] years; 228 [81.7%] male; 131 [46.9%] from centers in Asia; 101 [36.2%] nonviral cause; 173 [62.0%] extrahepatic disease), 37 were complete responders. The median progression-free survival and overall survival of complete responders was not reached with a median follow-up duration of 24 months (Figure 1A and 1B). At 24 months, the proportion of patients with no evidence of disease progression was 58.0% (95% CI, 36.2%-74.7%) among complete responders and 7.3% (95% CI, 3.5%-13.1%) among other patients. At 24 months, 81.1% (95% CI, 64.4%-90.5%) of complete responders and 21.2% (95% CI, 15.6%-27.3%) of other patients remained alive. Cox regression analyses adjusting for age, sex, race, α-fetal protein (AFP), liver disease cause, and extrahepatic disease confirmed that overall survival was greater in complete responders compared with other patients when accounting for clinical factors (hazard ratio [HR], 0.22; 95% CI, 0.07-0.70).

Similar results were observed in the multicenter cohort, where 17 complete responders were identified from 194 patients (mean [SD] age, 64.3 [11.4] years; 155 male patients [78.7%]; 94 [48.5%] from centers in Asia; 76 [39.2%] nonviral cause; 80 [41.2%] extrahepatic disease). The median progression-free survival and overall survival of complete responders was not reached with a median follow-up period of 29 months (Figure 1C and 1D). At 24 months, the proportion of patients with no evidence of disease progression was 87.4% (95% CI, 58.2%-96.7%) among complete responders and 19.4% (95% CI, 11.6%-28.7%) among other patients. At 24 months, 93.3% (95% CI, 61.2%-99.0%) of complete responders and 25.5% (95% CI, 16.2%-35.8%) of nonresponders remained alive. Cox regression analyses adjusting for age, sex, race, AFP, Child-Pugh class, liver disease cause, and extrahepatic disease confirmed that overall survival was greater in complete responders compared with other patients when accounting for clinical factors (HR, 0.06; 95% CI, 0.01-0.44). The median duration of immunotherapy treatment among complete responders in the multicenter cohort was 24 months, with 15 patients still without evidence of disease.

**Figure 2.**
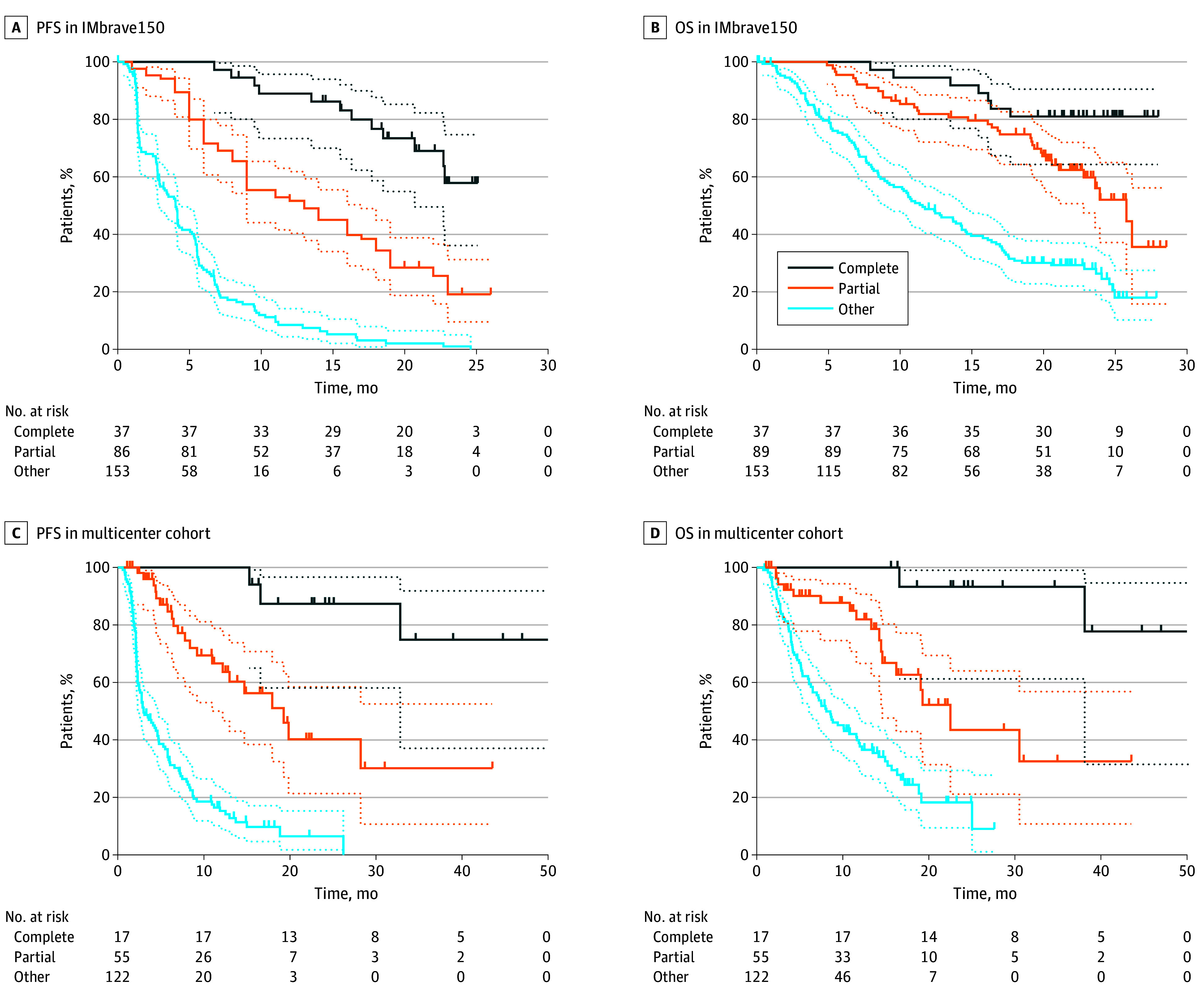
Survival Outcomes Among Complete Responders, Partial Responders, and Nonresponders (Stable and Progressive Disease) to Immunotherapy A and B, Graphs show Kaplan-Meier analyses of progression-free survival (PFS) (A) and overall survival (OS) (B) in the IMbrave150 cohort. C and D, Graphs show Kaplan-Meier analyses of PFS (C) and OS (D) in the multicenter cohort. Dotted lines indicate 95% CIs.

In the IMbrave150 cohort, patients who attained a partial response, defined as a greater than 30% reduction in tumor lesion sizes but not meeting criteria for a complete response, had longer progression-free (HR, 0.32; 95% CI, 0.23-0.41) and overall (HR, 0.36; 95% CI, 0.26-0.5) survival compared with patients without objective responses (Figure 2A and 2B). Partial responders had a worse progression-free (HR, 3.4; 95% CI, 2.14-5.48) and overall (HR, 2.67; 95% CI, 1.42-5.01) survival compared with patients with complete responses (Figure 2A and 2B). Similarly, in the multicenter cohort, patients who attained a partial response had longer progression-free (HR, 0.24; 95% CI, 0.17-0.34) and overall (HR, 0.33; 95% CI, 0.22-0.49) survival compared with patients without objective responses (Figure 2C and 2D). Partial responders in the multicenter cohort had worse progression-free (HR, 5.24; 95% CI, 2.33-11.75) and overall (HR, 6.74; 95% CI, 2.73-16.67) survival compared with patients with complete responses (Figure 2C and 2D).

**Figure 3.**
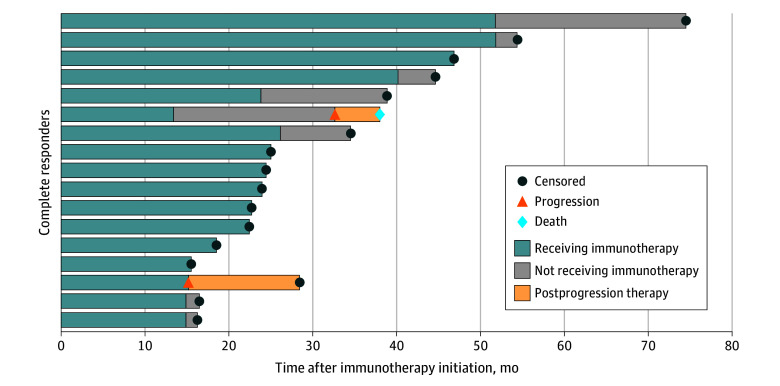
Duration of Treatment and Postprogression Outcomes Among Immunotherapy Complete Responders Treatment duration, duration not receiving treatment, and survival time after progression are depicted in a swimmer plot for complete responders in the multicenter cohort.

Among complete responders in the multicenter cohort, 7 patients electively discontinued immunotherapy, and a single patient subsequently had recurrence of disease 19 months later that failed to respond to a rechallenge of immunotherapy (Figure 3). No disease recurrence was observed in the remaining patients who electively discontinued immunotherapy, including 1 patient who was disease-free 23 months after stopping treatment (Figure 3).

**Figure 4.**
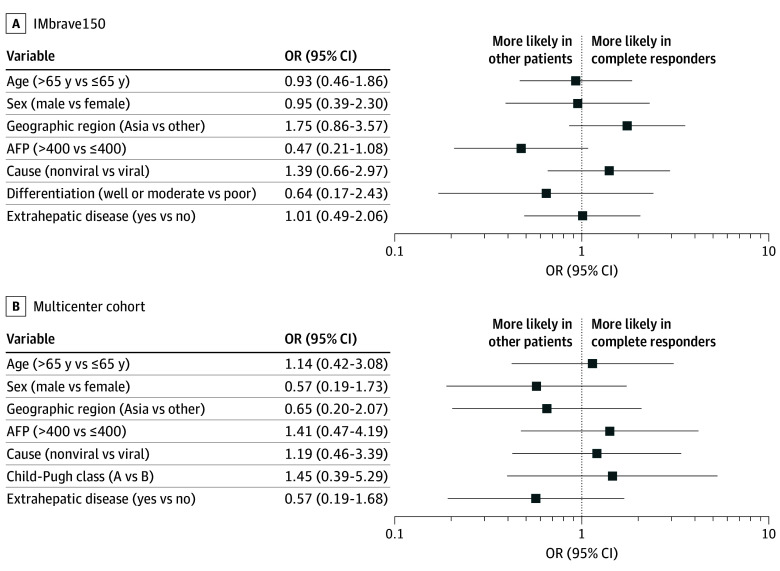
Clinical Characteristics of Immunotherapy Complete Responders and Other Patients Odds ratios (ORs) of the association between demographics and other clinical attributes in complete responders compared with other patients in the IMbrave150 cohort (A) and in the multicenter cohort (B) are shown. Error bars depict 95% CIs. AFP indicates α-fetal protein.

Across the IMbrave150 and multicenter cohorts, complete responders had a median (IQR) age of 67 (61-72) years and were predominantly male. Comparisons of the demographics of complete responders with those of other patients showed no statistically significant differences in patient characteristics (Figure 4A and 4B). Other clinical characteristics and pathologic factors that may influence prognosis, including race, body mass index, viral cause, AFP, extrahepatic disease, and tumor grade, were also indistinguishable between complete responders and other patients in both the IMbrave150 and multicenter cohorts (Figure 4A and 4B).

**Figure 5.**
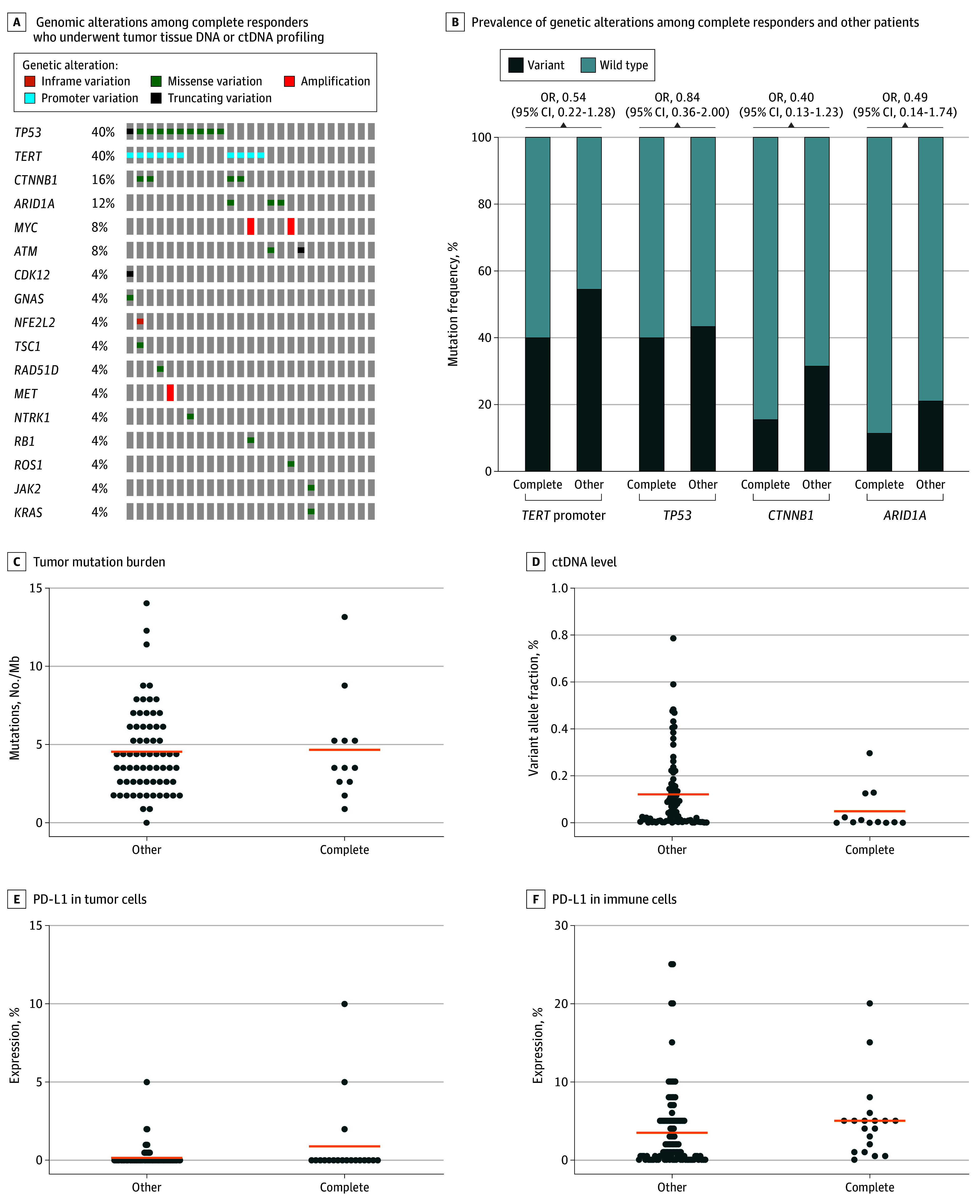
Genomic and Programmed Cell Death 1 Ligand 1 (PD-L1) Protein Expression Analyses of Immunotherapy Complete Responders and Other Patients A, Genomic alterations among 25 complete responders who underwent tumor tissue DNA or circulating tumor DNA (ctDNA) profiling. B, Bar charts indicate the prevalence of genetic alterations among complete responders and other patients. C, Tumor mutation burden assessed by targeted gene panel sequencing in complete responders and other patients (2-tailed *U* Mann-Whitney test; *P* = .91). D, The variant allele fraction of ctDNA was lower in complete responders vs other patients (2-tailed Mann-Whitney *U* test; *P* = .01). E, PD-L1 protein expression in tumor cells assessed by immunohistochemistry in complete responders and other patients (2-tailed Mann-Whitney *U* test; *P* = .74). F, PD-L1 protein expression in immune cells is increased in complete responders vs other patients (2-tailed Mann-Whitney *U* test; *P* = .04). Mb indicates megabase; OR, odds ratio.

A subset of 85 patients in the IMbrave150 cohort underwent targeted DNA sequencing on pretreatment tumor tissue, and 92 patients in the multicenter cohort underwent targeted DNA sequencing on pretreatment ctDNA.^8^ Owing to the relatively small sample size of complete responders with molecular profiling data across the entire study (35 patients), patients from both cohorts were pooled in subsequent analyses to determine whether genetic features may be associated with complete response. The most common genetic alterations in complete responders involved the *TERT* promoter (40%), *TP53* (40%), *CTNNB1* (16%), and *ARID1A* (12%) (Figure 5A). No genetic alterations were significantly associated with complete response (Figure 5B). Tumor mutation burden was also indistinguishable between complete responders and other patients (Figure 5C). However, complete responders exhibited significantly lower levels of ctDNA compared with other patients (Figure 5D).

In the IMbrave150 cohort, immunohistochemical analysis of PD-L1 protein expression was performed in a subset of patients with available pretreatment tissue. PD-L1 protein expression was assessed in either the tumor or immune cell compartment, which showed a statistically significant enrichment of PD-L1 protein expression in immune cells but not tumor cells (Figure 5E and 5F).

## Discussion

Understanding long-term treatment outcomes of immunotherapy in HCC is critical for guiding clinical decisions and may provide insights to tailor treatments for individual patients. In this cohort study, our post hoc assessment of the IMbrave150 trial and analysis of a contemporary multicenter population highlights how complete responders represent a nontrivial proportion of patients who are biologically distinct given their highly durable responses and survival. Patients who attained a partial response had a greater progression-free and overall survival benefit compared with patients who only attained stable or progressive disease, but their outcomes were still inferior to those of complete responders. Thus, the depth of immunotherapy response is likely associated with long-term outcomes in HCC, which supports the examination of additional strategies of using immunotherapies in combinations with locoregional therapy, such as with neoadjuvant or adjuvant treatment.^9,10,11^ Given that complete responders also experienced disease control even after the cessation of immunotherapy, our results suggest that indefinite treatment is not necessary in some patients with advanced stage disease. Nonetheless, the optimal length of immunotherapy treatment for advanced stage HCC and the utility of other modalities, such as liver transplantation, after a complete response remain to be defined.^12^

Stage migration after initiation of systemic therapies is not frequently encountered, but the nontrivial prevalence of durable complete responders brings into question how such patients should be managed according to existing treatment algorithms, such as the Barcelona Clinic Liver Cancer system.^13^ It remains unknown whether durable complete responders have attained a functional or actual cure, but further research may help ascertain whether such patients may be considered for curative intent modalities, such as liver transplantation for patients with only hepatic disease or resection for evaluation of pathologic response.

Our analysis indicates that complete responders do not have unique clinical characteristics, including demographics or established prognostic factors, such as disease cause, tumor differentiation, and AFP levels. Thus, adverse factors conventionally associated with worse outcomes may not affect the ability of immunotherapy to induce complete and durable tumor responses. We also did not identify specific genomic alterations associated with complete response. However, pretreatment ctDNA levels were diminished in complete responders, suggesting that ctDNA may represent a novel predictive marker for immunotherapy treatment in HCC. In addition, although PD-L1 protein expression has not been consistently associated with immunotherapy outcomes in HCC, we identified that PD-L1 protein expression in immune cells was associated with complete response.^4,8,14^ Further studies are needed to corroborate our results given the relatively small sample size of patients in this study with molecular profiling data, but these findings suggest that ctDNA and immune PD-L1 protein expression may be used as stratification markers of tumors with exquisite and prolonged sensitivity to immunotherapies.

### Limitations and Strengths

This study is limited by the relatively small sample size of complete responders, which is attributable to the rarity of such tumor responses to immunotherapy. In particular, this may have reduced the statistical power of this study to detect associations between complete response and molecular features, given that only a subset of patients in both cohorts underwent genetic and immunohistochemical profiling. In addition, longer-term follow-up is necessary to elucidate patterns of progression and postprogression treatment outcomes among complete responders.

Nonetheless, the results of this study are likely generalizable and relevant to other patient populations as they were consistent across a pivotal trial population and a multicenter patient cohort. In addition, this study provides novel clinical and biological insights into the mechanisms underlying complete responses to immunotherapy in HCC, which underscores the importance of continued investigation into the long-term effects and durability of immunotherapy, and may inform clinical practice by identifying potential biomarkers for response, guiding patient selection, and optimizing treatment strategies.

## Conclusions

In this post hoc trial and multicenter cohort analysis of patients with HCC treated with immunotherapy, complete responders demonstrated prolonged survival and durable disease control even after discontinuation of therapy. Biological features of complete responders were also distinct, and further evaluation of immune cell PD-L1 protein expression and ctDNA as potential biomarkers is warranted.

## Notes

Supplement 1. **eFigure.** CONSORT diagram of IMbrave150 analysis cohort

Supplement 2.
Data Sharing Statement
