# Supplementary material for: Complete Response to Immunotherapy in Patients With Hepatocellular Carcinoma
Source: JAMA Netw Open. 2025 Feb 25;8(2):e2461735. doi: 10.1001/jamanetworkopen.2024.61735 (PMC11862977; doi:10.1001/jamanetworkopen.2024.61735)
Supplement: Supplement 1. — eFigure. CONSORT diagram of IMbrave150 analysis cohort [file jamanetwopen-e2461735-s001.pdf]

Supplemental Online Content

Lim M, Espinoza M, Huang YH, Franses J, Zhu H, Hsiehchen D. Complete response to immunotherapy in patients with hepatocellular carcinoma. *JAMA Netw Open*. 2025;8(2):e2461735. doi:10.1001/jamanetworkopen.2024.61735

eFigure. CONSORT diagram of IMbrave150 analysis cohort

This supplemental material has been provided by the authors to give readers additional information about their work.

© 2025 Li M et al. *JAMA Network Open*.

eFigure. CONSORT diagram of IMbrave150 analysis cohort.

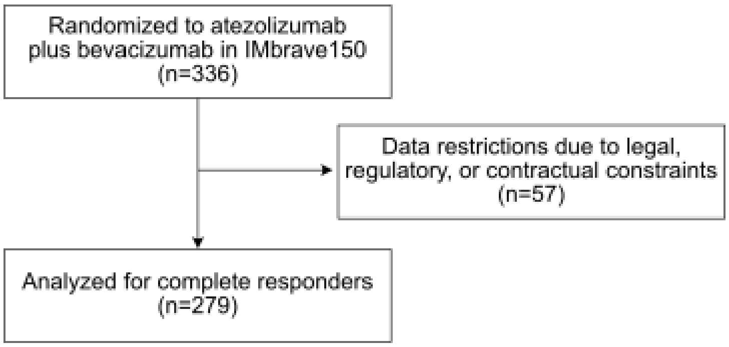

© 2025 Li M et al. *JAMA Network Open*.
