# Supplementary material for: Complete Response to Immunotherapy in Patients With Hepatocellular Carcinoma
Source: JAMA Netw Open. 2025 Feb 25;8(2):e2461735. doi: 10.1001/jamanetworkopen.2024.61735 (PMC11862977; doi:10.1001/jamanetworkopen.2024.61735)
Supplement: Supplement 2. — Data Sharing Statement [file jamanetwopen-e2461735-s002.pdf]

Data Sharing Statement

Lim. Complete Response to Immunotherapy in Patients With Hepatocellular Carcinoma. *JAMA Netw Open*. Published February 25, 2025. doi:10.1001/jamanetworkopen.2024.61735

Data
Data available: No
